# Prognostic significance of nuclear RNA export factor 3 in hepatocellular carcinoma

**DOI:** 10.3892/ol.2014.1809

**Published:** 2014-01-16

**Authors:** JIA-HAO JIANG, QIANG GAO, AI-WU KE, YAO YU, GUO-MING SHI, JIA FAN, JIAN ZHOU, XIAO-WU HUANG

**Affiliations:** 1 Key Laboratory of Carcinogenesis and Cancer Invasion, Liver Cancer Institute, Zhongshan Hospital, Fudan University, Ministry of Education, Shanghai 200032, P.R. China; 2 Shanghai Key Laboratory of Organ Transplantation, Shanghai 200032, P.R. China

**Keywords:** hepatocellular carcinoma, nuclear RNA export factor 3, nucleocytoplasmic transport, prognosis

## Abstract

Studies have highlighted important features of the nucleocytoplasmic transport of mRNAs and proteins. Nuclear RNA export factor 3 (NXF3) is a member of the nuclear RNA export factor family that plays a role in mediating the export of cellular mRNA from the nucleus to the cytoplasm for translation. However, little is known about the clinical significance of NXF3 in human tumors. To evaluate the prognostic significance of NXF3 in hepatocellular carcinoma (HCC), the expression levels of NXF3 in a cohort of 112 patients with primary HCC who had undergone hepatectomy for histologically confirmed HCC were assessed by immunohistochemistry. It was identified that the expression levels of NXF3 were higher in the primary HCC tissues compared with those in paired peritumoral liver tissues. The overexpression of NXF3 in the HCC tissues was correlated with decreased survival time [hazard ratio (HR) = 1.954, 95% confidence interval (CI) = 1.034–3.695, P=0.039] and earlier tumor recurrence (HR = 2.101, 95% CI = 1.186–3.722, P=0.011) in postoperative patients with HCC. Notably, overexpression of NXF3 was correlated with a poor survival time and increased recurrence following HCC resection in male patients (P=0.020 and P=0.007, respectively) but not in female patients (P=0.916 and P=0.821, respectively). In conclusion, the findings provide evidence that implicates NXF3 as a prospective predictor of HCC prognosis as well as a potential therapeutic target for cancer treatment.

## Introduction

Hepatocellular carcinoma (HCC) is the second most common cause of male cancer-related mortalities worldwide, with ~700,000 cancer mortalities due to HCC were reported in 2008 (1). Despite progress in early diagnosis and surgical interventions, the long-term survival rate of patients with HCC remains unsatisfactory due to the high rate of recurrence and metastasis (2). Thus, it is important to explore prognostic factors for HCC and to develop effective therapeutic schemes.

Studies have highlighted important features of the nucleocytoplasmic transport of RNAs and proteins (3). A number of the transport factors have been demonstrated to be dysregulated in primary tumor specimens and linked to poor prognosis (4,5). Nuclear RNA export factor 3 (NXF3) is a member of the nuclear RNA export factor (NXF) family of proteins, which plays a role in mediating the export of cellular mRNA from the nucleus to the cytoplasm for translation (6). NXF3 has been identified as a nucleocytoplasmic shuttle protein and demonstrated to induce RNA export by recruitment of Crm1, which is overexpressed in various types of human cancer (6,7). A study has shown that NXF3 may mediate the downregulation of the levels of transforming growth factor β3 (TGF-β3) mRNA expression and protein secretion in Sertoli cells (8), and TGF-β3 is considered to be involved in tumor progression (9). However, the roles of NXF3 in human tumor development and/or progression remain undetermined and no association between NXF3 and the clinical significance of tumors has been established. In the present study, the expression levels and the clinical relevance of NXF3 were investigated in a cohort of 112 patients with primary HCC. To the best of our knowledge, this is the first time such a study has been conducted. The aim was to establish the association between NXF3 and HCC and identify novel prognostic factors for HCC.

## Materials and methods

### Study population

Immunohistochemistry (IHC) was performed on tumor and peritumoral tissue samples from 112 patients who had undergone curative resection for HCC at Zhongshan Hospital, Fudan University (Shanghai, China) between February and September 2005. All patients without distant metastasis or any form of anticancer treatment prior to the surgical resection were selected on the basis of complete clinicopathological and follow-up data for the patients. The clinical typing of tumors followed the tumor-node-metastasis (TNM) classification system of the American Joint Committee on Cancer and the Union for International Cancer Control (7th edition) (10). The histological grade of tumor differentiation was assigned using the Edmondson grading system (11). Ethical approval was obtained from the Zhongshan Hospital Research Ethics Committee and written informed consent was obtained from all patients.

### Follow-up after surgery

Patient follow-up was completed in March, 2010 with a median observation time of 48.7 months. A diagnosis of recurrence was based on the typical imaging appearance of hepatic lesions in computed tomography and/or magnetic resonance imaging scans at elevated α-fetoprotein (AFP) levels. The overall survival (OS) time was defined as the interval between surgery and when the patient succumbed to the disease or between surgery and the last observation for surviving patients. Time to recurrence (TTR) was defined as the interval between the date of surgery and that of recurrence. The data were censored at the last follow-up for living patients and patients without any sign of recurrence.

### Evaluation of immunohistochemical findings

Tissue sections (4 μm) were stained with hematoxylin and eosin for histological analysis and with specific primary anti-human antibodies against NXF3 (1:150; LS-C31687; LifeSpan Biosciences, Inc., Seattle, WA, USA) for IHC. Following microwave antigen retrieval, the tissues were incubated with the primary antibodies overnight at 4°C followed by a 30-min incubation with the secondary antibody (Dako EnVision kit, Dako, Glostrup, Denmark). The reaction was visualized with diaminobenzidine and the tissues were counterstained with hematoxylin.

The tissue sections were viewed at ×200 magnification using a Leica DMI6000B inverted microscope (Leica Microsystems, Heidelberg, Germany) and images were captured. Two experienced pathologists independently assessed all IHC staining. The scoring for nuclear NXF3 expression was based on the staining proportion and intensity. The staining proportion was scored as follows: 0–25% staining, 1; 26–50% staining, 2; 51–75% staining, 3; and 76–100% staining, 4. The staining intensity was scored as follows: Negative intensity, 0; weakly positive, 1; moderately positive, 2; and strongly positive, 3, according to a previous study (12). The sum of the proportion and intensity scores was used to calculate the final staining score, which was then categorized as low (1–5) or high (6–7).

### Statistical analysis

Statistical analysis was performed with SPSS software, version 17.0 (SPSS, Inc., Chicago, IL, USA). The cumulative survival time was calculated using the Kaplan-Meier method and analyzed with the log-rank test. Univariate and multivariate analyses were performed based on the Cox proportional hazards regression model. The Student’s t-test and χ^2^ test were used as appropriate. P<0.05 was considered to indicate a statistically significant difference.

## Results

### NXF3 expression in HCC tissues

**Figure 1:**
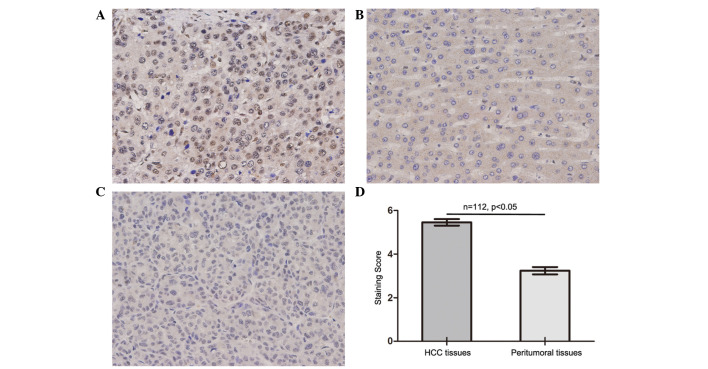
Identification of the NXF3 protein by immunohistochemical staining in representative HCC and paired non-tumor tissues. (A) High and (B) low intratumoral levels of NXF3 in HCC tissues; and (C) the adjacent non-tumor tissues. (D) A significant difference in the staining score of the tumor tissues and adjacent non-tumor tissues (P<0.001; paired samples t-test). Magnification, ×400. HCC, hepatocellular carcinoma; NXF3, nuclear RNA export factor 3.

NXF3 staining was mainly observed in the nucleus of the tumor cells. Significantly higher expression levels of NXF3 were identified in the HCC tissues compared with those in the paired peritumoral liver tissues, with intense expression of NXF3 in the cancerous tissues and weak expression in the normal liver tissues (P<0.001, Fig. 1). High expression levels of NXF3 in the HCC tissues were prevalent in the patients, and were observed in 78 (~70%) of the total 112 patients (Table I).

### Clinical relevance of NXF3 expression in HCC

**Figure 2:**
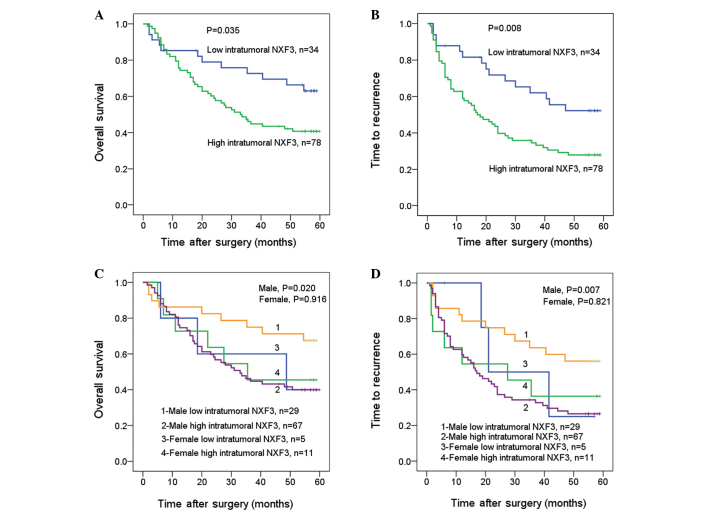
Kaplan-Meier analysis of the survival of patients with high or low intratumoral levels of NXF3. (A and B) Patients with high intratumoral levels of NXF3 had a poorer OS time or TTR than the patients with low intratumoral levels of NXF3. (C and D) High intratumoral levels of NXF3 were associated with a poor prognosis for OS time and TTR in male cases. NXF3, nuclear RNA export factor 3; OS, overall survival; TTR, time to recurrence.

Table I summarizes the clinicopathological data of the patients in the present study. The one-, three- and five-year overall and recurrence-free survival rates of the 112 patients were 81.2, 53.9 and 47.2% and 69.3, 42.6 and 35%, respectively. The univariate analysis revealed that the AFP levels, γ-glutamyltransferase levels, tumor size and TNM stage were correlated with the OS time and TTR, and that the tumor differentiation and vascular invasion were correlated with the OS time rather than TTR (Table II). Notably, the univariate analysis also revealed that individuals with high intratumoral expression levels of NXF3 had a significantly worse prognosis than those with low intratumoral expression levels of NXF3, as reflected by the OS and TTR (P=0.035 and P=0.008, respectively; Fig. 2A and B). The median OS and TTR for patients with low intratumoral expression levels of NXF3 were 55 and 42 months, respectively, as compared with 33 and 17 months for patients with high intratumoral expression levels of NXF3. Notably, high intratumoral expression levels of NXF3 correlated with a poor prognosis in men (P=0.020 and P=0.007 for the OS and TTR, respectively), while the prognostic value in women requires further analysis with a larger number of samples since no significant correlation was identified (P=0.916 and P=0.821 for the OS and TTR, respectively) (Fig. 2C and D). It may be concluded that intratumoral NXF3 represents a promising prognostic variable for the prediction of HCC pathogenesis, particularly in male patients with HCC.

To further confirm the prognostic significance of NXF3 expression levels, Cox multivariate proportional hazards regression analysis was performed with all the variables that were identified as significantly associated with the OS and/or TTR in the univariate analysis to control for confounders. The multivariate analysis showed that the patients with high intratumoral expression levels of NXF3 were 2.68- and 2.79-fold more likely to succumb to the disease [95% confidence interval (CI) = 1.28–5.60, P=0.009] and experience recurrence (95% CI = 1.44–5.37, P=0.002; Table III), respectively, than those with low intratumoral expression levels of NXF3. Other characteristics, including the AFP levels, γ-glutamyltransferase levels, tumor size, tumor differentiation, vascular invasion and TNM stage, were also assessed and the results are presented in Table III.

## Discussion

The proteins of the NXF family play roles in the transport of mRNAs from the nucleus to the cytoplasm, which is fundamental for gene expression (13). Yang *et al* (6) demonstrated that the expression of NXF3 was tissue-specific, that NXF3 was detected at high levels in the testes and that the RNA export induced by NXF3 could be inhibited by leptomycin B (an antibiotic that specifically blocks Crm1 function), indicating that NXF3 is an adapter for Crm1-dependent nuclear mRNA export. Although Crm1 is overexpressed in various types of human cancer, including glioma, cervical cancer and renal cell carcinoma, and has the potential to be a prognostic marker for cancer (14–16), the clinical relevance of NXF3 in human cancer remains undetermined. In the present study, the data revealed that NXF3 expression levels were markedly elevated in primary human HCC tissues compared with those in peritumoral liver tissues. Furthermore, the clinically relevant data presented showed that patients with HCC and high tumor NXF3 expression levels had decreased OS times and earlier TTR compared with those of patients with low tumor NXF3 expression levels. These data indicate that NXF3 protein expression may be a promising prognostic biomarker for HCC and raises the possibility that NXF3 may play a role in promoting the transformation of hepatocytes to tumor cells, possibly by mediating the dysregulation of nucleocytoplasmic transport.

Additionally, NXF3 expression levels showed prognostic value in male patients with HCC but not in female ones. This finding may be due to the human X chromosome having the property that one X chromosome undergoes inactivation in females (17,18). This feature leads to a genetic situation unique to females in which mutations in oncogenes (possibly including NXF3) or tumor suppressor genes on the inactive chromosome are not expressed. As males have only one maternally inherited X chromosome, they are expected to be more susceptible to oncogenic alterations on the X chromosomes inherited from their mothers (19). Epidemiological data have revealed that the incidence of HCC shows a gender discrepancy, with males accounting for more than two-thirds of HCC cases worldwide (20,21). The factors that contribute to this gender discrepancy remain unclear. Sex hormones and environmental factors are considered to be important in the gender discrepancy in the process of hepatocarcinogenesis (22), while the present study indicates that the X-linked genes (based on data of NXF3) may also play a role in this gender discrepancy.

Nucleocytoplasmic transport maintains the balance of spatial regulation of protein activity and thus is critical for normal cell function (23). The transport of oncogenes and tumor suppressors is disrupted in various types of cancer cells. The forkhead box O (FOXO) transcription factors, including FOXO1a, FOXO3a and FOXO4, are located in the cell nucleus and negatively regulate cell growth, proliferation, differentiation and survival (24). The inactivation of these factors may be induced by inappropriate nuclear export and cytoplasmic mislocalization that is mediated by Crm1 and contributes to the development of glioblastoma multiforme, renal cancer and colon cancer (23,25). Any change in the subcellular localization of oncoproteins and tumor suppressors has the potential to affect the regulation and activity of FOXO transcription factors. Based on the theory that a variety of vital proteins are mislocalized in cancer cells, strategies for redirecting these proteins to the correct subcellular location may be developed to provide effective cancer therapies (26). Therefore, such factors (including Crm1 and NXF3) that are involved in RNA or protein export may yield novel therapeutic targets for cancer treatment.

Although a number of target proteins, including p53, p21, FOXO and NF-κB, have been identified that undergo nuclear export in a Crm1-dependent manner (27), few mRNAs or proteins for NXF3-dependent nuclear export have been identified thus far. A study has shown that NXF3 mediates the downregulation of the levels of TGF-β3 mRNA expression and protein secretion in Sertoli cells (8), but additional studies are required to confirm whether TGF-β3, which is considered to be involved in tumor progression, may be a candidate target gene mediated by NXF3-dependent nuclear export. In the present study, the findings provide a preliminary connection between NXF3 expression levels and HCC. Further studies are required to identify the specific mRNAs or proteins for NXF3-dependent nuclear export and to establish the exact role of NXF3 in the pathogenesis of HCC.

In summary, NXF3, as an NXF family member, has for the first time, to the best of our knowledge, been demonstrated to be linked to human cancer, extending its role into tumor development. Furthermore, NXF3 was identified to be correlated with the overall and recurrence-free survival time in postoperative patients with HCC, suggesting that NXF3 may be a promising prognostic marker for HCC as well as a novel RNA export pathway for targeting with cancer therapies.

## Figures and Tables

**Table I:** Summary of the clinicopathological data of the 112 patients in the NXF3 protein expression study.

Variable	Cases, n (%)
Gender	
Male	96 (85.7)
Female	16 (14.3)
Age (years)	
≤51	58 (51.8)
>51	54 (48.2)
HBsAg	
Negative	13 (11.6)
Positive	99 (88.4)
AFP (ng/ml)	
≤20	42 (37.5)
>20	70 (62.5)
GGT (U/l)	
≤54	56 (50)
>54	56 (50)
Liver cirrhosis	
No	19 (17.0)
Yes	93 (83.0)
Tumor size (cm)	
≤5	55 (49.1)
>5	57 (50.9)
Tumor number	
Single	100 (89.3)
Multiple	12 (10.7)
Tumor encapsulation	
Complete	72 (64.3)
None	40 (35.7)
Tumor differentiation	
I–II	84 (75.0)
III–IV	28 (25.0)
Vascular invasion	
No	69 (61.6)
Yes	43 (38.4)
TNM stage^a^	
I	61 (54.5)
II–III	51 (45.5)
NXF3 expression	
Low	34 (30.4)
High	78 (69.6)

a The clinical typing of tumors followed the TNM classification system of the American Joint Committee on Cancer and the Union for International Cancer Control (edition 7).

NXF3, nuclear RNA export factor 3; HBsAg, hepatitis B surface antigen; AFP, α-fetoprotein; GGT, γ-glutamyltransferase; TNM, tumor-node-metastasis.

**Table II:** Univariate analyses of factors associated with HCC survival and recurrence.

	OS			TTR		
		
Variable	HR	95% CI	P-value	HR	95% CI	P-value
Age (years; ≤51 vs. >51)	1.185	0.707–1.984	0.520	1.392	0.871–2.224	0.166
Gender (female vs. male)	0.909	0.446–1.850	0.791	0.996	0.510–1.944	0.990
HBsAg (negative vs. positive)	1.106	0.475–2.576	0.816	1.055	0.506–2.202	0.886
AFP (ng/ml; ≤20 vs. >20)	2.520	1.397–4.546	0.002	2.385	1.418–4.010	0.001
GGT (U/l; ≤54 vs. >54)	2.362	1.380–4.042	0.002	2.400	1.481–3.889	<0.001
Liver cirrhosis (no vs. yes)	1.683	0.764–3.710	0.196	1.750	0.869–3.523	0.117
Tumor size (cm; ≤5 vs. >5)	1.766	1.042–2.993	0.034	1.615	1.006–2.593	0.047
Tumor number (single vs. multiple)	1.127	0.511–2.485	0.767	1.405	0.697–2.832	0.342
Tumor encapsulation (complete vs. none)	1.207	0.685–2.126	0.514	1.366	0.854–2.208	0.203
Tumor differentiation (I–II vs. III–IV)	1.865	1.111–3.131	0.018	1.263	0.758–2.105	0.370
Vascular invasion (no vs. yes)	2.112	1.260–3.540	0.005	1.586	0.992–2.537	0.054
TNM stage (I vs. II–III)	2.201	1.307–3.707	0.003	1.892	1.185–3.021	0.008
Intratumoral NXF3 (low vs. high)	1.954	1.034–3.695	0.039	2.101	1.186–3.722	0.011

Univariate analysis, Cox proportional hazards regression model. HCC, hepatocellular carcinoma; OS, overall survival time; TTR, time to recurrence; HR, hazard ratio; CI, confidence interval; HBsAg, hepatitis B surface antigen; AFP, α-fetoprotein; GGT, γ-glutamyltransferase; TNM, tumor-node-metastasis; NXF3, nuclear RNA export factor 3.

**Table III:** Multivariate analyses of factors associated with HCC survival and recurrence.

Survival	HR	95% CI	P-value
OS			
AFP (ng/ml; ≤20 vs. >20)	2.451	1.316–4.563	0.005
GGT (U/l; ≤54 vs. >54)	1.887	1.041–3.423	0.036
Tumor size (cm; ≤5 vs. >5)	1.318	0.720–2.415	0.371
Tumor differentiation (I–II vs. III–IV)	0.771	0.413–1.441	0.416
Vascular invasion (no vs. yes)	2.205	0.817–5.951	0.118
TNM stage (I vs. II–III)	1.111	0.417–2.962	0.833
Intratumoral NXF3 (low vs. high)	2.680	1.282–5.603	0.009
TTR			
AFP (ng/ml; ≤20 vs. >20)	2.088	1.234–3.534	0.006
GGT (U/l; ≤54 vs. >54)	1.745	1.030–2.958	0.039
Tumor size (cm; ≤5 vs. >5)	1.419	0.835–2.413	0.196
TNM stage (I vs. II–III)	1.771	1.073–2.924	0.025
Intratumoral NXF3 (low vs. high)	2.785	1.444–5.372	0.002

Multivariate analysis, Cox proportional hazards regression model. HCC, hepatocellular carcinoma; HR, hazard ratio; CI, confidence interval; OS, overall survival time; AFP, α-fetoprotein; GGT, γ-glutamyltransferase; TNM, tumor-node-metastasis; TTR, time to recurrence.
